# UHRF2 promotes Hepatocellular Carcinoma Progression by Upregulating ErbB3/Ras/Raf Signaling Pathway

**DOI:** 10.7150/ijms.60030

**Published:** 2021-06-26

**Authors:** Jingjie Sun, Kejia Wu, Siyuan Chen, Shiming Jiang, Yong Chen, Changzhu Duan

**Affiliations:** 1 Department of Cell Biology and Genetics, Chongqing Medical University, #1 Yixueyuan Road, Chongqing 400016, China.; 2 Department of Hepatobiliary Surgery, The First Affiliated Hospital of Chongqing Medical University, Chongqing Medical University, Chongqing 400000, China.

**Keywords:** UHRF2, hepatocellular carcinoma, progression, ErbB3

## Abstract

Emerging evidence revealed that UHRF2 was implicated in a variety of human diseases, especially in cancer. However, the biological function, clinical significance and underly mechanisms of UHRF2 in hepatocellular carcinoma (HCC) is largely unknown. We analyzed the expression of UHRF2 in 371 HCC tissues and 50 para-cancerous tissues of TCGA database. We found that UHRF2 was significantly upregulated in HCC tissues, which was further confirmed in HCC cells and tissues by western blot. More importantly, the level of UHRF2 was correlated with pathological grade and clinical stage, and the patients with high level of UHRF2 had lower overall survival, disease-free survival and higher recurrence rate than those with low UHRF2 level. Univariate and multivariate Cox regression analysis revealed that high level of UHRF2 might be an independent prognostic factor for HCC patients. Functional investigations suggested that ectopic expression of UHRF2 could promote the proliferation, migration and invasion of HCC cell lines, whereas knock down of UHRF2 exhibited an opposite effect. Additionally, gene set enrichment analysis indicated that ERBB signaling pathway was upregulated in patients with high level of UHRF2. Pearson correlation analysis indicated that the expression of UHRF2 was positively correlated with ErbB3 and its downstream targets SOS1, Ras and Raf-1. Furthermore, we found that overexpression of UHRF2 could upregulate the expression of ErbB3, SOS1, Ras and Raf-1. Our findings suggested that UHRF2 might accelerate HCC progression by upregulating ErbB3/Ras/Raf signaling pathway and it might serve as a diagnostic marker and therapeutic target for HCC patients.

## Introduction

Hepatocellular carcinoma (HCC), as the most common primary liver cancer, accounts for about 80% of primary liver tumors 1. It has been reported that HCC leads to estimated 700,000 deaths every year and has become the third most leading cause of cancer-related death worldwide 1, 2. HCC is an aggressive disease with a poor prognosis, which typically develops on the background of chronic liver disease that driven by the interaction of environmental factors, genetic predisposition and viral infection, especially chronic HBV and HCV infection 3. Since the clinical manifestations of HCC occur at the advanced stage, the early diagnosis and effective treatment of HCC is still a challenge.

Ubiquitin-Like with PHD and ring finger domains 2 (UHRF2), a member that belongs to the family of UHRF, contains five recognizable functional domains, namely the ubiquitin-like domain (UBL) domain, tandem-Tudor domain (TTD), plant homeodomain (PHD), SET and RING associated (SRA) domain, and really interesting new gene (RING) finger domain 4. Due to the complex structure, UHRF2 possesses multiple functions in diverse cellular processes. As a ubiquitin E3 ligase, UHRF2 could ubiquitinate PCNP, a nuclear protein that contains two remarkable PEST sequences which are rich in proline (P), glutamic acid (E), serine (S), and threonine (T) 5, 6. It has been also reported that UHRF2 could serve as a vital cell cycle regulator by interacting with multiple cyclins, CDKs, p53, pRB and PCNA 6. UHRF2 has been revealed to possess epigenetic regulation function and is capable of maintaining 5mC levels in certain genomic loci in brain 7 and stabilizes TIP60 to regulate H3K9ac and H3K14ac through RING finger domain 8. Moreover, UHRF2 could promote DNA damage repair by reducing the level of p21 mediated by RING finger domain 9. Recently, emerging evidence indicated that UHRF2 was involved in the tumorigenesis and progression of several human cancers, such as esophageal squamous cell carcinoma, lung cancer and colorectal cancer 10-12. However, the biological role and clinical significance of UHRF2 in HCC have not yet been fully elucidated.

ErbB3 is a member of EGFR family of receptor tyrosine kinases, which serves as a homolog to EGFR (ErbB1) and HER2 (ErbB2) and plays an important role in cancers 13. ErbB3 was upregulated in various cancers, such as breast cancer, gastric cancer, ovarian cancer, prostate cancer, bladder cancer, colon cancer, neck squamous cell carcinoma and melanoma 14-24. Previous studies reported that the high level of ErbB3 and the activation of its downstream signaling including PI3K/AKT pathway and Ras/Raf pathway participated in the development and progression of cancers 25. Up to now, the relationship between UHRF2 and ErbB3 is unclear.

In this study, we found that UHRF2 was obviously increased in HCC tissues compared with normal tissues. The subsequent analyses showed that high level of UHRF2 was significantly correlated with the malignant characteristics of HCC and it could predict poor prognosis of patients. Further functional investigations and bioinformatics analyses revealed that UHRF2 could promote the proliferation and invasion of HCC by upregulating ErbB3/Ras/Raf signaling.

## Materials and methods

### Patient samples

The 20 pairs of HCC tissues and adjacent non-tumor tissues were collected from the patients who were diagnosed with HCC and had undergone hepatectomy at the First Affiliated Hospital of Chongqing Medical University (Chongqing, China). All the patients did not suffer from other malignancy or receive any preoperative chemotherapy or radiotherapy. The samples of each patient were obtained following informed consent and stored in liquid nitrogen immediately after surgical resection. The study was approved by the Ethics Committee of Chongqing Medical University and conducted in accordance with the Declaration of Helsinki.

### Data set and Gene set enrichment analysis (GSEA)

The level 3 gene expression profile of 371 HCC tissues and 50 non-cancerous tissues and corresponding clinical data were downloaded from The Cancer Genome Atlas (TCGA) data portal (https://www.cancer.gov/about-nci/organization/ccg/research/structural-genomics/tcga). The study was performed in accordance with the publication guidelines of TCGA. As the data were obtained from TCGA, no ethics committee approval was further needed. GSEA was performed with GSEA 4.0.3 (https://www.gsea-msigdb.org/gsea/index.jsp) in KEGG gene sets.

### Cell culture

Human HCC cell lines (HepG2.2.15, Hep AD38, HepG2 and Huh7) and normal hepatic cell line HL-7702 were purchased from American Type Culture Collection (ATCC, Manassas, VA, USA). All these cell lines were cultured in DMEM (HyClone, UT, USA) supplemented with 10% fetal bovine serum, 100 mg/mL streptomycin and 100 U/mL penicillin in a humidified incubator at 37 °C with 5% CO_2_.

### Vector construction, siRNAs and cell transfection

To overexpress UHRF2 in HCC cells, the full-length sequence of UHRF2 was synthesized and then inserted into the Flag tagged vector pcDNA3.1-3×Flag, named as Flag-UHRF2. The empty Flag tagged vector with no UHRF2 sequence served as control and named as Flag. The vectors were finally verified by sequencing. To knock down UHRF2, two siRNAs (si-UHRF2#1 and si-UHRF2#2) against UHRF2 and a siRNA-NC were synthesized by Ribobio (Guangzhou, China). The vectors for transfection were extracted with Plasmid Mini Kit I (Norcross, GA, USA). Cell transfection were performed with Lipofectamine^TM^ 2000 (Thermo Fisher Scientific, Waltham, MA, USA) according to the manufacturer's instruction.

### qRT-PCR

Total RNA of HCC cells was exacted with Trizol (Takara, Dalian, China) and reversely transcribed into cDNA using PrimeScript RT Reagent Kit (Takara, Dalian, China) according to the manufacturer's protocols. PCR amplifications were performed with TB Green Premix Ex Taq (Takara, Dalian, China) in a Bio-Rad CFX96 system (Bio-Rad, CA, USA). The relative expression of UHRF2 was calculated with 2^-ΔΔCT^ method and normalized to the internal reference GAPDH.

### Western blot analysis

The treated HCC cells were washed three times with PBS and lysed with RIPA buffer. The protein was obtained and denatured with 5×loading buffer at 100℃ for 10 min, and separated by 10% SDS-PAGE, then transferred to PVDF membranes (Bio-Rad, CA, USA). The membranes were blocked with 5% skimmed milk and then incubated with primary antibodies against UHRF2 (1:1000), p-Raf (1:1000) (Abcam, Burlingame, CA, USA), Ras (1:1000) or Raf (1:1000) (Bioworld Technology, Inc., USA) at 4℃ overnight and then incubated with secondary antibodies (1:5000) at room temperature for 2 h. The bands were detected with Immobilob™ Western Chemiluminescent HRP Substrate (Millipore, Billerica, MA, USA).

### Cell proliferation assays

The proliferation of HCC cells was detected with Cell Counting Kit-8 (CCK-8, Bosterbio, Wuhan, China) and Cell-Light™ EdU DNA Cell Proliferation Kit (Ribobio, Guangzhou, China) in accordance with the manufacturer's protocols, respectively. For colony formation assays, HCC cells (2000/well) were seeded into 6-well plates and cultured for 10 days. The cells were washed 3 times with PBS and fixed with 4% paraformaldehyde for 20 min and followed by staining with crystal violet for 10 min. The stained cells were photographed with a microscope (Leica, Wetzlar, Germany).

### Cell migration and invasion assays

HCC cells were seeded into 6-well plates and then scratched with a 200 μL pipette tip in the middle of the wells and cultured with serum-free medium for 36 h. The scratch widths of each group at 0 h and 36 h were photographed with a microscopy (Leica, Wetzlar, Germany). The relative migration of HCC cells was quantified with Image J. As to invasion assays, 4×10^5^ HCC cells were suspended with serum-free medium and seeded in the upper chambers covered with matrigel (BD Biosciences, NJ, USA). And the lower chambers were added with 500 μL complete medium. After 24 h, the cells on the upper chambers were removed with cotton swabs, and the cells on the lower chambers were fixed with 4% paraformaldehyde for 20 min and stained with crystal violet for 10 min. The stained cells were counted under a microscopy (Leica, Wetzlar, Germany).

### Statistical analysis

Statistical analyses were conducted with SPSS 19.0 (IBM, SPSS, Chicago, IL, USA). The graphs were created with GraphPad Prism 5. Differences between groups were analyzed with Student's t test, one-way ANOVA or χ^2^ test. The survival rates and recurrence rate were assessed by Kaplan-Meier method and tested by log-rank test. The correlation between continuous variables was evaluated by Pearson correlation. Univariate and multivariate Cox proportional hazards regression were applied to identify the risk factors associated with the survival of HCC patients. The receiver operating characteristic (ROC) curve was used to evaluate the diagnostic value. All of the statistical analyses were considered as statistically significant when *P<*0.05.

## Results

### UHRF2 is highly expressed in HCC and associated with poor prognosis

**Figure 1:**
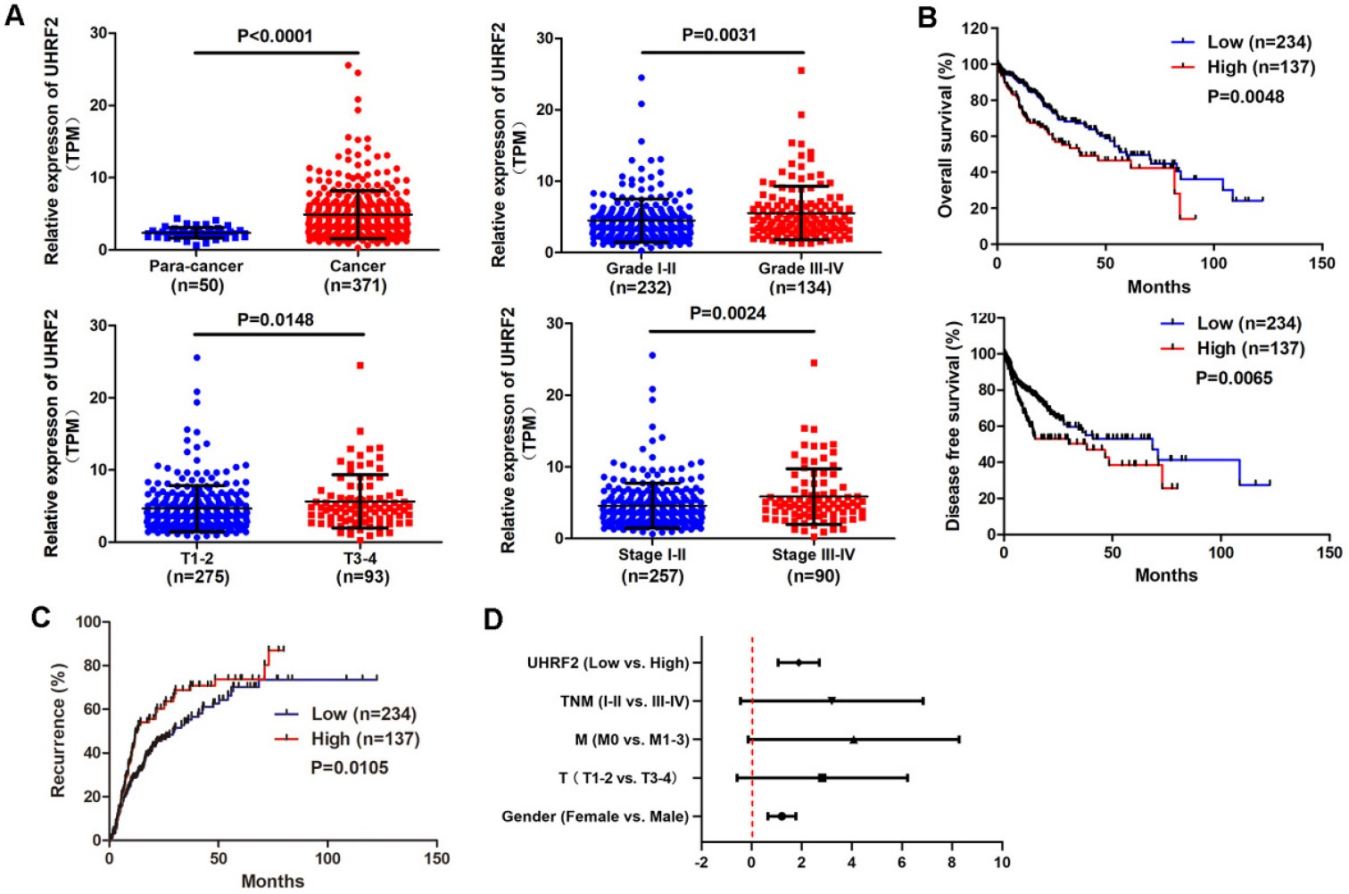
UHRF2 is highly expressed in HCC and associated with poor prognosis. **(A)** The expression of UHRF2 in HCC tissues (n=371) and para-cancerous tissues (n=50) and in different pathological grade, T stage and clinical stage. **(B)** Kaplan-Meier survival curves showing the correlation between the level of UHRF2 and overall survival and disease-free survival time. (**C**) Kaplan-Meier recurrence curve showing the correlation between the level of UHRF2 and recurrence rate of HCC patients. The HCC patients were categorized into high expression group and low expression group by mean expression of UHRF2. **(D)** Univariate and multivariate Cox regression analyses of factors associated with overall survival of HCC patients.

In order to understand the expression of UHRF2 in HCC, the gene expression profile of 371 HCC tissues and 50 para-cancerous tissues were downloaded from TCGA database. The expression of UHRF2 was analyzed and it was found that UHRF2 was significantly upregulated in HCC tissues compared with para-cancerous tissues (**Fig. 1A**). The correlation between UHRF2 expression and clinical characteristics of these HCC patients were analyzed and listed in **Table 1**. Patients with higher pathological grade, T stage or TNM stage exhibited higher expression of UHRF2 (**Fig. 1A**). Kaplan-Meier survival curves indicated that the HCC patients with higher expression of UHRF2 had a shorter overall survival time and disease-free survival time than those with lower UHRF2 level (**Fig. 1B**). Further recurrence analysis indicated that the level of UHRF2 was positively correlated with the recurrence rate of HCC patients (**Fig. 1C**). Univariate and multivariate Cox regression analyses showed that high UHRF2 level could be an independent prognostic factor for the patients with HCC (**Fig. 1D**).

### UHRF2 protein level is upregulated in HCC cell lines and tissues

**Figure 2:**
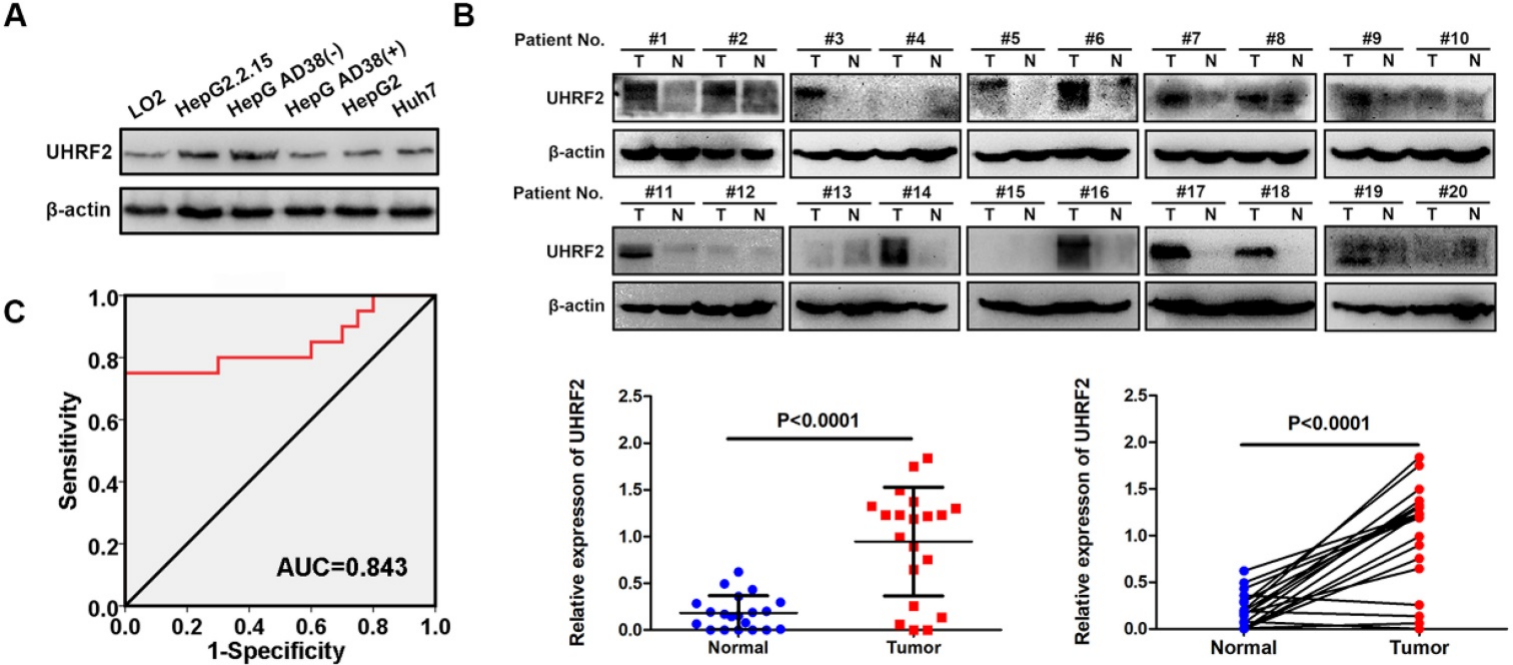
UHRF2 protein level is upregulated in HCC cell lines and tissues.** (A)** The protein level of UHRF2 in normal hepatic cell line LO2 and HCC cell lines HepG2.2.15 and Hep AD38(-). **(B)** The protein level of UHRF2 in 20 pairs of HCC tissues and para-cancerous tissues. **(C)** Diagnostic value of UHRF2 for HCC was analyzed by ROC curve.

The protein level of UHRF2 was then detected by western blot in 20 pairs of HCC tissues, HCC cell lines (HepG2.2.15, Hep AD38, HepG2 and Huh7) and normal hepatic cell line HL-7702. UHRF2 protein level was also highly expressed in HCC tissues and cell lines compared with para-cancerous tissues and normal hepatic cell line (**Fig. 2A-B**). The relationship between UHRF2 protein level and clinical characteristics of the 20 HCC patients were listed in **Table S1**. The level of UHRF2 was correlated with TNM stage of HCC patients. The ROC curve showed that UHRF2 could sensitively discriminate HCCs from normal tissues (**Fig. 2C**). These results indicated that UHRF2 might play a crucial role in HCC, we therefore focused our attention on UHRF2 in the tumorigenesis and progression of HCC.

### UHRF2 promotes the proliferation, migration and invasion of HCC cell lines

**Figure 3:**
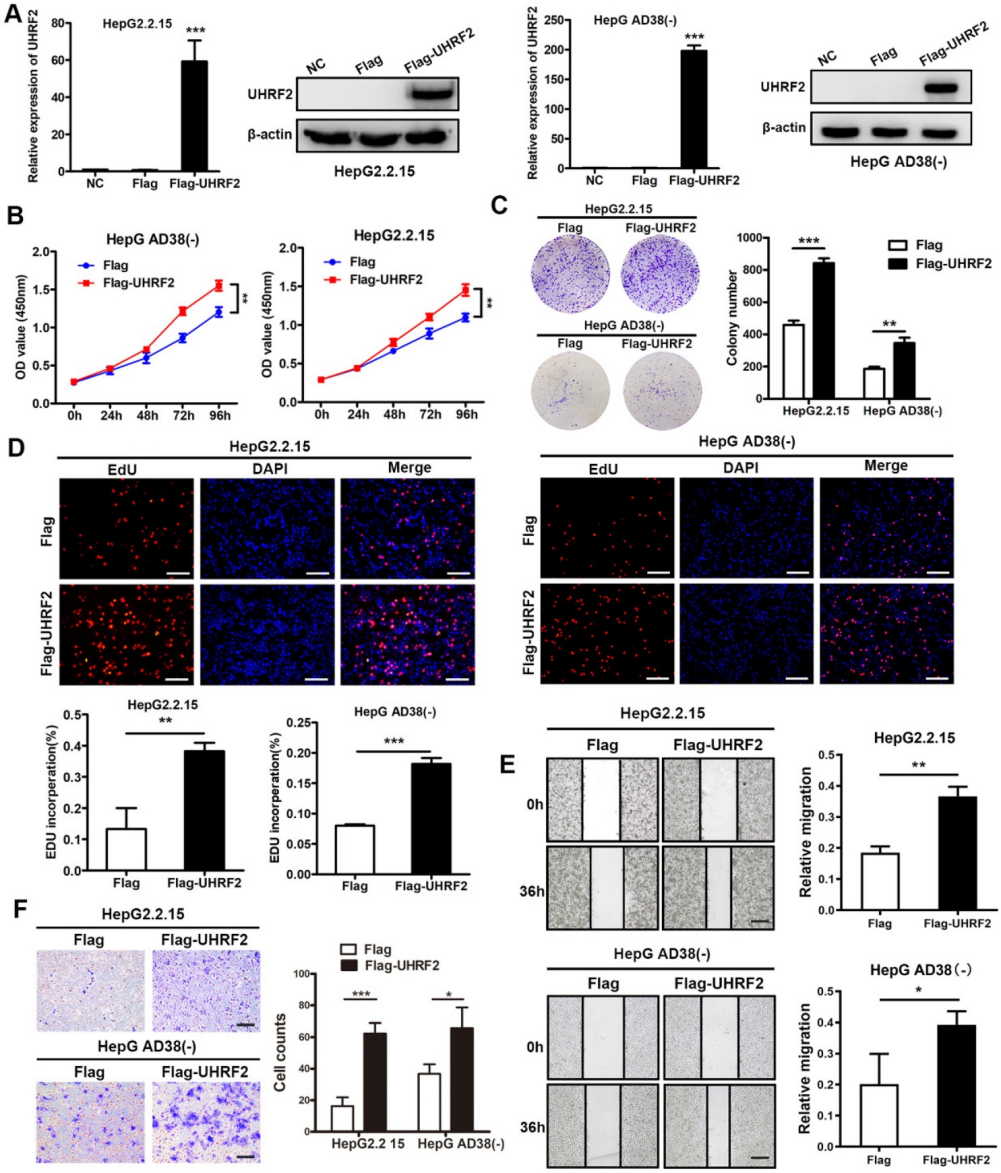
Overexpression of UHRF2 promotes the proliferation, migration and invasion of HCC cell lines. **(A)** The expression of UHRF2 was detected by qRT-PCR and western blot in HepG2.2.15 and Hep AD38(-) cells after transfected with Flag or Flag-UHRF2. **(B)** Growth curves of HepG2.2.15 and Hep AD38(-) cells were determined by CCK-8 assays. (**C**) Colony formation assays were performed to evaluate the proliferation of HepG2.2.15 and Hep AD38(-) cells. (**D**) EdU assays were conducted in HepG2.2.15 and Hep AD38(-) cells. Scale bar, 100 μm. (**E**) Wound healing assays were applied to detect the migration of HepG2.2.15 and Hep AD38(-) cells. Scale bar, 200 μm. (**F**) Transwell assays were used to determine the invasion abilities of HepG2.2.15 and Hep AD38(-) cells. Scale bar, 100 μm. Data were showed as mean ± SD. *P< 0.05, **P<0.01, ***P<0.001.

To investigate the biological function of UHRF2 in HCC, the overexpression vector and siRNAs of UHRF2 were constructed. We then transfected HepG2.2.15 and Hep AD38(-) cells with these vectors and siRNAs and assessed the overexpression and knock down efficiency by qRT-PCR and western blot. The results showed that UHRF2 was successfully overexpressed or knocked down at both mRNA and protein level in HCC cell lines by these vectors and siRNAs (**Fig. 3A and Fig. S1A**). The proliferation of HCC cells was determined by CCK-8 assays, which revealed that overexpression of UHRF2 markedly promoted cell growth, whereas knock down of UHRF2 exhibited an opposite effect (**Fig. 3B and Fig. S1B**). Colony formation assays showed that the colony number in UHRF2 overexpression group was notably more than control group (**Fig. 3C**). Additionally, 5-ethynyl2' deoxyuridine (EdU) assays indicated that ectopic expression of UHRF2 significantly increased the percentages of EdU-positive cells (**Fig. 3D**). Wound healing and Transwell assays were further applied to evaluate the effect of UHRF2 on HCC cell migration and invasion. The results indicated that the cell motility of HCC cells was enhanced by overexpression of UHRF2 and impaired by downregulation of UHRF2 (**Fig. 3E-F and Fig. S1C-D**). These findings suggested that UHRF2 could promote the proliferation and motility of HCC cells.

### UHRF2 promotes HCC progression by upregulating ErbB3/Ras/Raf signaling pathway

**Figure 4:**
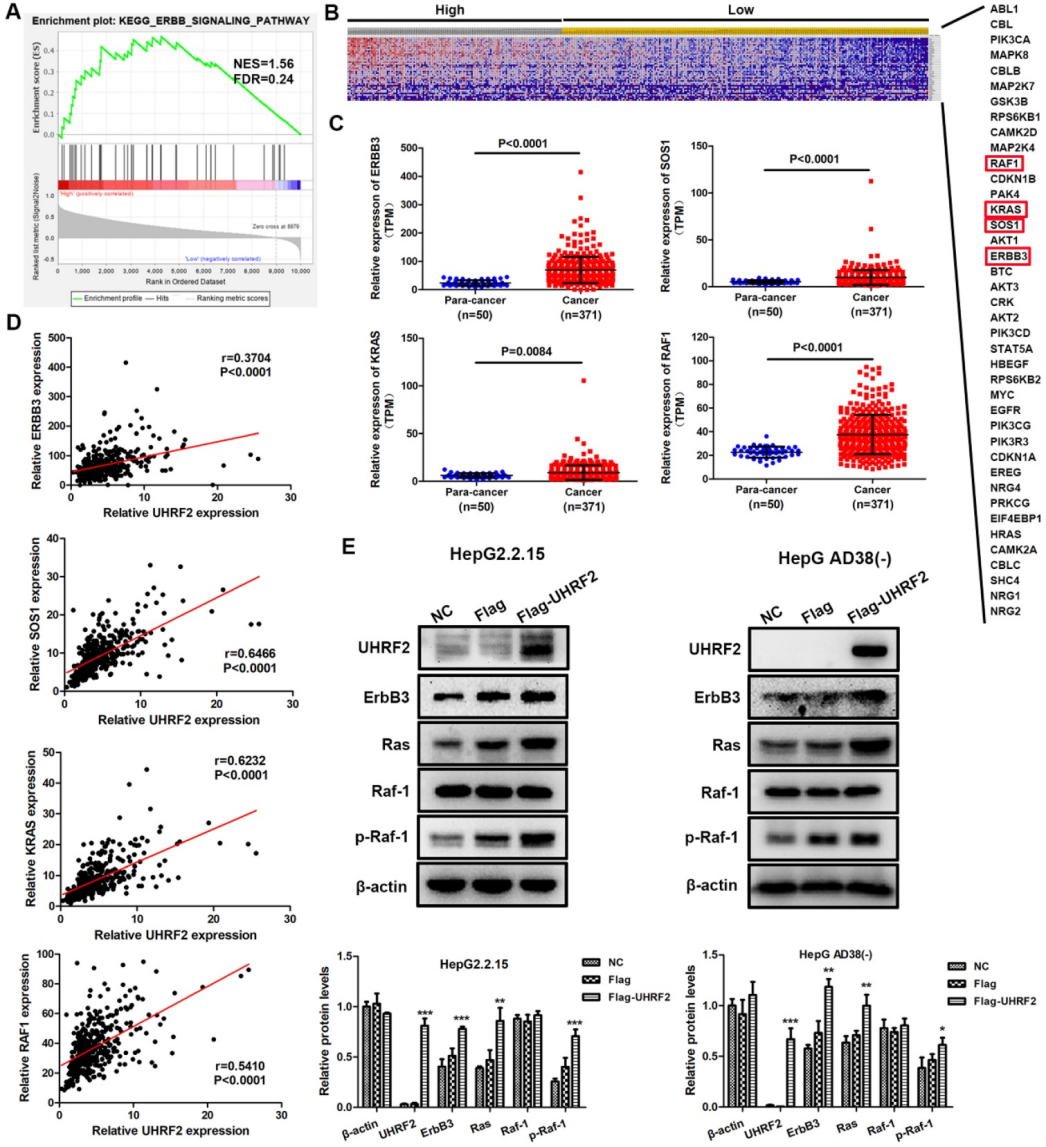
UHRF2 promotes HCC progression by upregulating ErbB3/Ras/Raf signaling pathway. **(A)** GSEA showed that ERBB signaling pathway was upregulated in UHRF2 high expression group. The HCC patients were categorized into high expression group and low expression group by mean expression of UHRF2. **(B)** Heatmap indicated the expression of 40 genes involved in ERBB signaling pathway in 371 HCC patients. **(C)** The expression of ErbB3, SOS1, Ras and Raf-1 in 371 HCC tissues and 50 para-cancerous tissues. **(D)** Pearson correlation analyses between the expression of UHRF2 and ErbB3, SOS1, Ras and Raf-1 in 371 HCC tissues. **(E)** The expression of ErbB3, Ras and Raf-1 was detected by western blot in HepG2.2.15 and Hep AD38(-) cells.

To elucidate how UHRF2 affects the biological behaviors of HCC cell lines, the HCC cases of TCGA database were divided into high and low expression group by mean expression of UHRF2 and GSEA was then carried out with the expression profile of these HCC cases in KEGG gene sets. The results showed that ERBB signaling pathway was significantly upregulated in UHRF2 high expression group with a normalized enrichment score (NES) of 1.555 and a P value of 0.026 (**Fig. 4A**). Meanwhile, a total of 40 protein coding genes involved in ERBB signaling pathway were identified, which were depicted by heatmap (**Fig. 4B**). Among these genes, it was found that the expression of ErbB3 and its downstream molecules SOS1, Ras and Raf-1 were all highly expressed in HCC tissues compared with non-cancerous tissues (**Fig. 4C**). Moreover, Pearson correlation analysis indicated that the expression of UHRF2 was positively correlated with ErbB3, SOS1, Ras and Raf-1 in HCC, respectively (**Fig. 4D**). Therefore, we supposed that UHRF2 might upregulate ErbB3/Ras/Raf signaling pathway to promote the progression of HCC.

To further verify the correlation between UHRF2 and ErbB3/Ras/Raf signaling in HCC, the HepG2.2.15 and Hep AD38(-) cells were transfected with UHRF2 overexpression vectors or siRNAs and followed by western blot. The results indicated that the expression of ErbB3 was significantly increased in UHRF2 overexpression group and decreased in UHRF2 knock down group (**Fig. 4E and Fig. S2A**). And as expected, the levels of phosphorylated Ras and Raf-1 displayed the same trend with UHRF2 (**Fig. 4E and Fig. S2**). These findings suggest that ErbB3 and its downstream Ras/Raf signaling pathway are essential for UHRF2 mediated progression of HCC.

## Discussion

HCC is the most prevalent primary liver cancer with a high morbidity and low 5-year survival rate 26, 27. It has been considered as a serious health problem that brings huge economic burden and psychological pressure to patients 28. Over the last decades, much progress has been made in prevention strategies, surveillance program, early diagnosis and treatment of HCC 29, 30. The major treatments including hepatic resection, liver transplantation and tumor ablation are potentially curative but a sufficient early stage of diagnosis is needed for the achievement of the best therapeutic effectiveness 31. However, a large proportion of HCC patients are diagnosed at an advanced stage often so that those treatment approaches above are not appropriate options for these patients 31, 32. Thus, identification of the biomarkers that participated in the progression of HCC might be beneficial to discover new prognostic markers and therapeutic targets for HCC patients.

In our study, we found that the expression of UHRF2 was much higher in HCC tissues compared with normal tissues based on the TCGA dataset. And the expression of UHRF2 was significantly correlated with pathological grade, T stage and TNM stage of HCC patients. Survival analyses demonstrated that the expression of UHRF2 was negatively associated with overall survival and disease-free survival rate and positively correlated with recurrence rate of HCC patients. Univariate and multivariate Cox regression analysis indicated that UHRF2 level could be an independent prognostic factor for HCC patients. To confirm the protein level of UHRF2 in HCC, 20 pairs of HCC tissues and adjacent non-cancerous tissues were randomly collected and analyzed by western blot. We found that UHRF2 protein in HCC tissues was also significantly upregulated with a high diagnostic value. Consistent with our findings, former research reported that both mRNA and protein level of UHRF2 were upregulated in intrahepatic cholangiocarcinoma tissues and the overall survival rate of intrahepatic cholangiocarcinoma patients with low UHRF2 level was obviously higher than those with high UHRF2 level 33. These results suggested that UHRF2 level might be a diagnostic and prognostic marker for HCC patients.

Further functional investigations indicated that UHRF2 enhanced the proliferation, migration and invasion of HCC cells. Generally, UHRF2 was considered as a nuclear E3 ubiquitin ligase which was involved in cell cycle progression by interacting with a variety of key factors of cell cycle 6. It has been reported that UHRF2 could lead to G0/G1 phase arrest of cell cycle through ubiquitinating cyclins D1 and E1 and served as a candidate tumor suppressor in breast cancer and lung cancer 4, 34, 35. Another study revealed that overexpression of UHRF2 inhibited cell migration and invasion of non-small cell lung cancer and the level of UHRF2 was positively correlated with overall survival of patients 11. However, other studies indicated that UHRF2 was upregulated in colorectal cancer and was associated with poor prognosis of patients 12, 36, 37. Moreover, UHRF2 could enhance the migration and invasion ability of gastric cancer cell lines by regulating epithelial-mesenchymal transition (EMT) process 38. These researches supported our results that UHRF2 might play an oncogenic role in tumor. The different expression patterns of UHRF2 in cancers revealed the complexity of the function of UHRF2 and its importance in tumorigenesis.

To further understand the probable mechanism underlying UHRF2 in HCC, GSEA was then applied to identify the signaling pathways affected by UHRF2 in HCC. We found that a total of 31 KEGG gene sets were significantly upregulated in HCC tissues with high level of UHRF2, among which ubiquitin-mediated proteolysis, cell cycle, Wnt signaling pathway, TGF-beta signaling pathway, chronic myeloid leukemia and ERBB signaling pathway were cancer-related pathways. As expected, ubiquitin-mediated proteolysis was significantly enriched with a highest enrichment score since UHRF2 was a well-known E3 ubiquitin ligase 39, 40. In line with our results, previous study showed that UHRF2 occupied a central position in cell cycle network 5. It also has been demonstrated that UHRF2 served as a positive regulator of Wnt signaling pathway to promote intestinal tumorigenesis 12. Another research indicated that UHRF2 was associate with TGFβ in esophageal squamous cell carcinoma 10. These investigations supported our results of GSEA. However, the correlation between UHRF2 and ERBB signaling pathway in HCC has not been reported. We found that UHRF2 was positively correlated with the expression of ErbB3 and its downstream targets SOS1, Ras and Raf-1 rather than other ERBB receptors based on TCGA dataset. ErbB3 was frequently upregulated in various of human cancers, including cervical cancer 41, breast cancer 14, gastric cancer 42, colorectal Cancer 43 and ovarian cancer 44. ErbB3 level was correlated with worse prognosis of colorectal cancer patients 45. Recent study revealed that overexpression of ErbB3 promoted the tumorigenesis and angiogenesis of nasopharyngeal carcinoma 46. In the present work, we found that overexpression of UHRF2 could increase the level of ErbB3 and its downstream targets SOS1, Ras and Raf-1 in HCC cell lines. It suggested that ErbB3/Ras/Raf signaling cascades might be regulated by UHRF2.

Taken together, we demonstrated that UHRF2 was upregulated in HCC and its level was significantly correlated with the prognosis of HCC patients. Moreover, our results revealed that UHRF2 might promote the progression of HCC through upregulating the ErbB3/Ras/Raf signaling cascades. Although the precise interaction between UHRF2 and ERBB signaling pathway in HCC and the function of UHRF2 *in vivo* need to be further explored, our findings laid a foundation for the further study of UHRF2 in HCC and provided novel ideas and clues for the diagnosis and treatment of HCC.

## Supplementary Material

Supplementary figures and table.

## Figures and Tables

**Table 1:** Correlation between UHRF2 expression and clinicopathological features in 371 HCC patients

Characteristics	Total	UHRF2 in HCC		Chi-square	*P* value
		Low	High		
**Age**				0.391	0.532
<50	70	42	28		
≥50	300	192	108		
**Gender**				3.643	0.056
Male	250	166	84		
Female	121	68	53		
**Histological grade**				6.071	0.014*
1-2	232	158	74		
3-4	134	74	60		
**T**				5.415	0.020*
T1-2	275	182	93		
T3-4	93	49	44		
**N**				1.122	0.289
N0	252	160	92		
N1-3	4	1	3		
**AFP**				5.060	0.024*
<400	213	144	69		
≥400	65	34	31		
**Clinical stage**				7.138	0.008*
I- II	257	172	85		
III-IV	90	46	44		

*P<0.05.
