# Supplementary material for: UHRF2 promotes Hepatocellular Carcinoma Progression by Upregulating ErbB3/Ras/Raf Signaling Pathway
Source: Int J Med Sci. 2021 Jun 26;18(14):3097–105. doi: 10.7150/ijms.60030 (PMC8364466; doi:10.7150/ijms.60030)
Supplement: Supplementary file 1 — Supplementary figures and table. [file ijmsv18p3097s1.pdf]

Table S1. Relationship between UHRF2 protein level and clinicopathological features in 20 HCC patients.

Characteristic		UHRF2		*P* value
		Low	High	
**Age**	<50	4	2	0.628
	≥50	6	8	
**Tumor size**	<5	3	4	1.000
	⩾5	7	6	
**AFP**	<400	9	6	0.303
	⩾400	1	4	
**HBV DNA**	-	8	4	0.170
	+	2	6	
**HBsAg**	-	0	1	1.000
	+	10	9	
**TNM stage**	I- II	8	2	**0.023***
	III-IV	2	8	

*P<0.05

Fig. S1

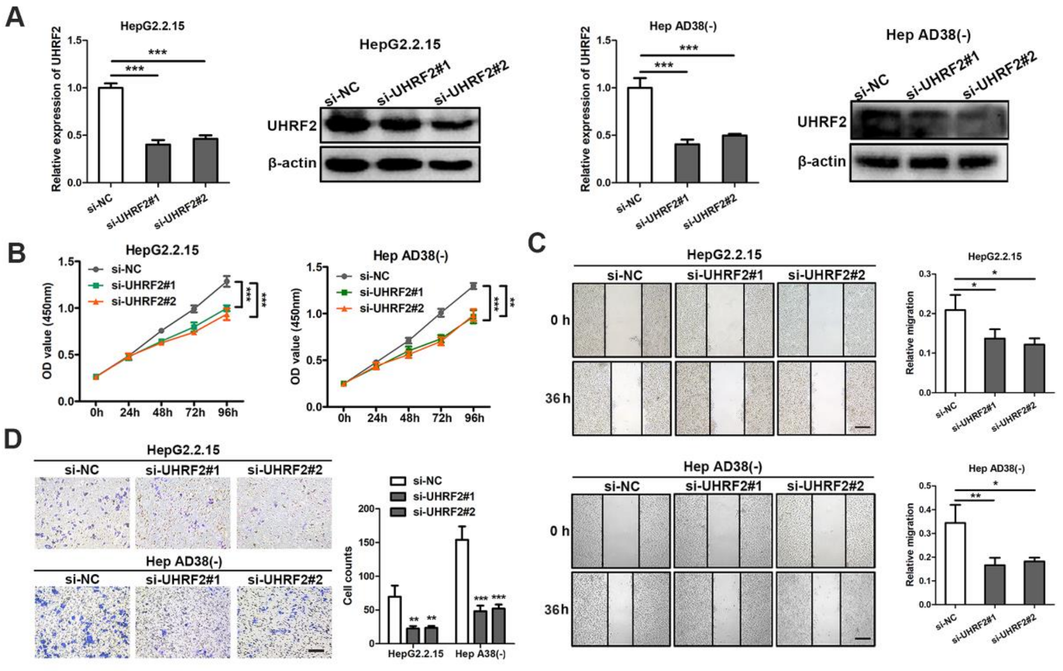
A
HepG2.2.15
Relative expression of UHRF2
si-NC
si-UHRF2#1
si-UHRF2#2
UHRF2
β-actin
Hep AD38(-)
B
OD value (450nm)
0h
24h
48h
72h
96h
C
0 h
36 h
Relative migration
D
Cell counts
HepG2.2.15
Hep A38(-)

Fig. S2

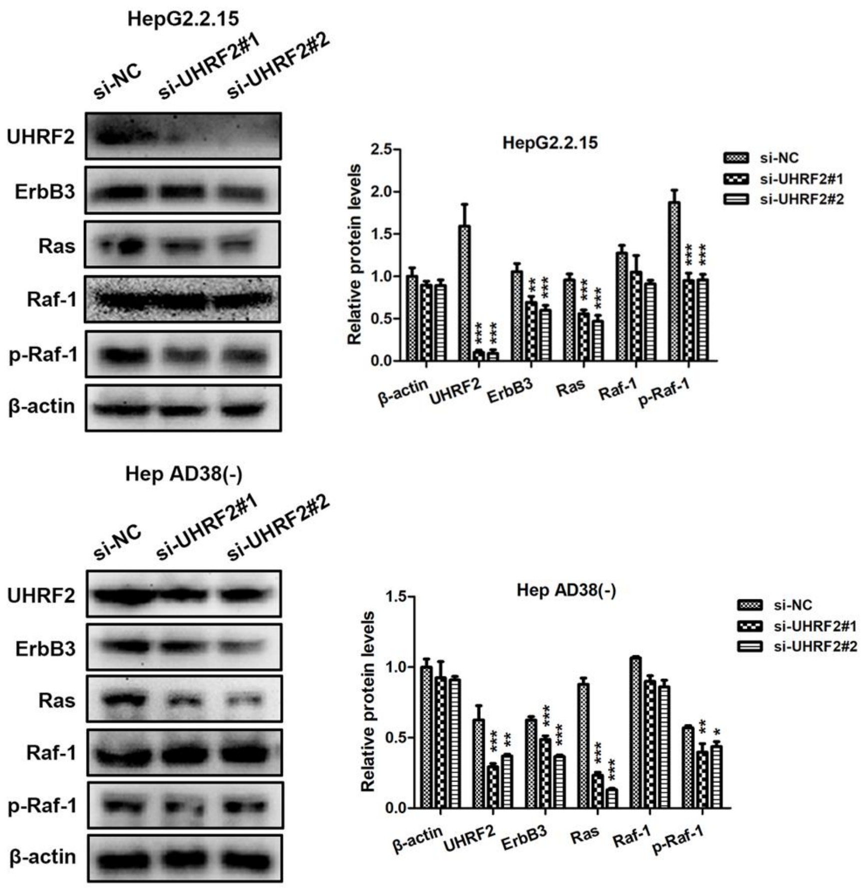
HepG2.2.15
si-NC
si-UHRF2#1
si-UHRF2#2
UHRF2
ErbB3
Ras
Raf-1
p-Raf-1
β-actin
HepG2.2.15
Relative protein levels
si-NC
si-UHRF2#1
si-UHRF2#2
β-actin
UHRF2
ErbB3
Ras
Raf-1
p-Raf-1
Hep AD38(-)
si-NC
si-UHRF2#1
si-UHRF2#2
UHRF2
ErbB3
Ras
Raf-1
p-Raf-1
β-actin
Hep AD38(-)
Relative protein levels
si-NC
si-UHRF2#1
si-UHRF2#2
β-actin
UHRF2
ErbB3
Ras
Raf-1
p-Raf-1

Figure S1. Knock down of UHRF2 inhibits the proliferation, migration and invasion of HCC cell lines. **(A)** The expression of UHRF2 was detected by qRT-PCR and western blot in HepG2.2.15 and Hep AD38(-) cells after transfected with indicated siRNAs. **(B)** Growth curves of HepG2.2.15 and Hep AD38(-) cells were determined by CCK-8 assays. **(C)** Wound healing assays were performed to detect the migration of HepG2.2.15 and Hep AD38(-) cells. Scale bar, 200 μm. **(D)** Transwell assays were applied to determine the invasion of HepG2.2.15 and Hep AD38(-) cells. Scale bar, 100 μm. Data were showed as mean ± SD. *P< 0.05, **P<0.01, ***P<0.001.

Figure S2. Downregulation of UHRF2 decreases the protein level of ErbB3/Ras/Raf signaling pathway. Data were showed as mean ± SD. *P< 0.05, **P<0.01, ***P<0.001.
